# Supplementary material for: Cost-efficient boundary-free surface patterning achieves high effective-throughput of time-lapse microscopy experiments
Source: PLoS One. 2022 Oct 27;17(10):e0275804. doi: 10.1371/journal.pone.0275804 (PMC9612557; doi:10.1371/journal.pone.0275804)
Supplement: Attachment — Submitted filename: Response to Reviewers.docx [file pone.0275804.s006.docx]

**Reviewer #1**: In this work, Liang et al. developed one simple method for high-throughput of time-lapse live cell experimental studies. In this method, the cells will be confined to high-adhesive zones through a cheap and boundary-free surface patterning protocol. This simple and cost-efficient method solved the cell-losing problem during time lapse microscopy movies and improved the effective-throughput experiments. Furthermore, the authors demonstrated that the ESC cell maintained their phenotype in a 4-day long experiment. Overall, this is a timely piece of study for live cell imagining studies, as cell loss is a big problem for long-time experiments. Here are some comments:
Response: We thank the reviewer for the positive comments on the value of the approach, methods, and results. We also appreciate the reviewer’s constructive comments and will address them point-by-point in the following.

1. Is it easier to do a simple experiment for the no-pattern situation and calculate the lineage recording rate?
Response: We thank the reviewer for the suggestion and agree that it is helpful to include the experimental observation as a comparison. We thus perform additional no-pattern experiment as suggested. Although the fact that cells always move out of the field of view makes it difficult to collect sufficient data for statistics, our observation, i.e., barely catch any entire lineage colony, confirms the no-pattern simulation result. More details are added in the Results and Discussion section.

2. The authors need to detail the imaging analysis for lineage tracking.
Response: We appreciate and agree with the suggestion. We have now added a new paragraph of imaging analysis details for lineage tracking in the Materials and Methods section.

3. The authors need to detail the simulation method for estimating the lineage recording rate in Fig. Response: We thank the reviewer for the suggestion. To better explain the method, we have now added a new paragraph of simulation method for estimating the lineage recording rate in the Materials and Methods section.

4. Fig. 3, why does the image on day 3 have a much brighter background than the others?
Response: We thank the reviewer for the comment. The different brightness is because we used different color contrast for the 405-fluorescent channel (the red shade). Although the 405 channel is only to show the location of patterns on the plate, we agree with the reviewer that this much brighter background can cause confusion. We have now re-adjusted the contrast in Figure 3 and achieved a more uniform background of 405 channel.

**Reviewer #2**: In this manuscript, Liang et al. describe a method that restricts cell migration to pre-defined boundary-free areas via differential cell-ECM affinities. The authors claim that this method is low-cost and that it significantly improves the throughput of time-lapse microscopy experiments by preventing cells from migrating out of the fields of view. The major caveat of this manuscript is that the authors do not provide sufficient benchmarks to demonstrate the advantages of their methods over the state-of-the-arts. Therefore, it remains difficult to evaluate whether their method provides a meaningful contribution to the community. Specifically, the authors should answer the following questions.
Response: We appreciate the reviewer’s constructive comments and agree with the reviewer that more comparison between state-of-the-arts with our method should be better clarified. We have now included additional interpretation as the reviewer suggested and will address both major and minor points in the following.

1. Does laminin and BSA coating alter cell phenotypes?
Factors such as ECM stiffnesses, local cell density, and the geometry of the culturing environment are known to regulate cell phenotypes. Therefore, a central task is to demonstrate that the coating does not significantly alter the cell phenotypes. While the authors demonstrated that the brachyury signals in ESCs on Day 4 matched the previous studies, this only reflects one aspect of cell phenotype in a highly specific experimental condition. The authors could perform parallel experiments in coated and uncoated plates and examine whether cell proliferation (e.g., quantify the intermitotic times by time-lapse microscopy or stain pRb S807/811 by IF) and the expression levels of stem-cell marker genes (by IF) are consistent. The authors should also consider comparing cells in the high-affinity areas, the low-affinity areas, and the transitory areas to demonstrate that the abrupt change in the cell-ECM affinity does not alter the cell phenotypes. Finally, benchmarking on other cell lines, such as cancer cells where ECM stiffnesses are known to regulate their therapeutic responses, will be useful. While it is understandable that no methodology is perfect, demonstrating the scope of applicability is necessary.
Response: We thank the reviewer for the comments and agree with the reviewer that many culturing environment factors will affect cell phenotypes.

In particular, we agree with the reviewer that, apart from Brachyury, we shall examine other cell proliferation factors, such as the intermitotic times. Thus, we have recorded ESCs’ division time on no-pattern laminin-coated surface versus patterned laminin/BSA-coated surface and found both cases have similar division duration. The results have now been included in the Results and Discussion section.

We also agree with the reviewer that, no methodology is perfect, demonstrating the scope of applicability is necessary. We have now added more details in Introduction, as well as Results and Discussion section, better clarify both the advantage and limitation of our method.

Although we really appreciate the reviewer’s constructive experimental suggestions about parallel experiments in uncoated plates for ESCs, this experiment can be practically challenging. The widely accessible/available time-lapse microscope imaging protocols, especially at the high amplification level, are mostly performed in glass-bottom plates. Up to our knowledge, ESCs are not able to grow on uncoated or low-affinity only coated glass. So, to achieve the comparison between different surface coating conditions (i.e., the purpose of parallel experiment), we perform an alternative experiment on the no-pattern no-BSA laminin-coated surface. As mentioned above, we have found the cells have similar division duration under both conditions.

2. Does this method provide similar performance to other state-of-the-art methods, such as photolithography, despite being cheaper? The authors could perform similar benchmarks described above in laminin/BSA-coated plates and photolithography-modified plates.
Response: We thank the reviewer for the suggestion. We have now added more detailed comparison between our system and the other state-of-the-art methods in Results and Discussion section.

3. Does this method allow one to track more cells throughout the imaging period?
In addition to the cells migrating out of the fields of view, the throughput of time-lapse microscopy experiments is governed by the accuracy of automatic cell tracking. As cells become more crowded with this method, the accuracy of segmentation (detecting cell nuclei) and track linking (mapping cell nuclei between images and detecting mitosis, etc) could decrease. It is thus necessary to track the cells cultured in coated and uncoated plates with standard automatic cell-tracking algorithms and examine the number of cells tracked throughout the imaging period and the accuracy of tracked cell lineages. If this method is only aimed at manual cell tracking, it should be clearly described.
Response: We appreciate the reviewer for the comments. We are sorry about the unclearness. We have now added a new paragraph of imaging analysis details for lineage tracking in the Materials and Methods section. As for the cell density, we did not see cell colony more crowded, probably because the patterned area is much larger than the cell size. To quantitatively prove the point, we have now compared the lineage tracking between pattern versus no-pattern situations and included the no-pattern time-lapse data in Supplementary Movie 4.

Minor Comments
1. The authors provide many technical details in the “Results and Discussion” section, such that it becomes too wordy. The authors could consider summarizing their findings in the main text and additionally providing a protocol with these technical details in the supplementary materials.
Response: We thank the reviewer for the comments. We have now summarized the findings in the main text and moved some of the technical details into the Materials and Methods section.

2. The authors should proofread their manuscripts to match the publication standard. For example, “250um” should be “250 μm” (notice the space between the number and the unit), and in the text “For the 35cm glass-bottom dish”, “35cm” should be “35 mm.”
Response: We thank the reviewer for the comments. We have added the space between all the number and the unit, further proofread the manuscript and made the modification to match the publication standard.

3 (Optional). The authors used a simple numeric simulation to demonstrate the improvement of the experimental throughputs. While it is effective, the authors could easily make the simulation more realistic, such as by simulating the cell division with the Gillespie algorithm. Alternatively, the authors could formulate their problem by calculating the first-passage time of an unbiased random walk, for which the closed-form solution exists.
Response: We thank the reviewer for the comments. To better apply the suggestions, we have invited a collaborator from Department of Mathematics, Prof. Jun Allard, as the co-author in this paper.

We together constructed a new simulation in the continuous stochastic growth mode by employing a Gillespie algorithm. The results are comparable to our previous synchronized cell cycle mode. We have now included both models in the paper, discussed the results in Results and Discussion, and added all the technical details into the Material and Methods.

Although we really appreciate the suggestions about the first-passage time, because the colony size is growing, up to our knowledge, there are no analytic solutions for the mean time to colony escape (i.e., the mean first-passage time; see non-closed-form progress on a similar problem in[1]), nor are there analytic solutions for the probability a given fraction of a colony will remain in the adhesive zone for all 4 days (corresponding to a survival probability, see Eq. 57 in [2]). As an alternative solution, we have made a rough estimate under the approximation that the colony size is fixed. Details are included in the Material and Methods.

1.	Simpson MJ, Baker RE. Exact calculations of survival probability for diffusion on growing lines, disks, and spheres: The role of dimension. J Chem Phys. 2015;143: 094109.

2.	Rupprecht J-F, Bénichou O, Grebenkov DS, Voituriez R. Exit time distribution in spherically symmetric two-dimensional domains. J Stat Phys. 2015;158: 192–230.
